# German Hospital Bath Houses

**Published:** 1898-08-20

**Authors:** 


### GERMAN HOSPITAL BATH HOUSES.

(From a Nurse Correspondent.)

Nurses on holiday invariably visit all the hospitals within their reach—a custom to be commended more from the professional than the health point of view. For the very essential of a holiday is to leave "shop" behind us, and clear out the cobwebs accumulated in the brain by a twelvemonth's ward routine. However, go to the hospitals I did on my tour of holiday-making in Germany, and found more novelty in the bath houses attached to them than I did in the wards.

Undoubtedly, baths are connected in the German, as in the Parisian mind, with a more or less diseased condition of the body. In German lodgings baths are by no means easy to get, and in many really good hotels the bath-rooms are both primitive and uninviting. No Continentals "tub" with the almost religious devotionalism practised by the enlightened Britisher, and to be guilty of regular and daily bathing in Germany, as in France and Italy, is sure to evoke the suspicion that one is suffering from a disease for which a bath cure has been prescribed, although sometimes it is referred to a curious British mania for cleanliness. Often, when asking a German landlady for a bath, she has inquired in turn whether I were ill, and unless one happens to take an inflatable rubber or travelling bath, ablutions abroad may have to be conducted under difficulties.

But the hospital bath houses are beautiful; many of them are most decoratively tiled and handsomely adorned, and the bath so clean and dainty that one is reminded of the descriptions one has read of the luxuries attendant on bathing in the East. In all the hospitals which I visited whilst in Germany I saw a great many cases of bed-sore treated by immersion in warm water, and the number of such patients set me wondering as to why so many cases should have been allowed to develop bed-sores. Many of the doctors said that water beds and air cushions are by no means so commonly in use in the German hospitals as with us, and to this and a less elaborate nursing system the prevalence of bed-sores must be referred. Each bath has a canvas hammock fixed, whereon the patient lies; a large square water cushion comfortably supporting the back, and water pillows packed conveniently under the head. The patient lies completely immersed, save for the head, and a wooden cover fits on the bath so that nothing can be seen of the patient excepting the head and face. The patients wear absolutely no clothing, not even a vest, so that whilst taking their meals and reading their paper there is a certain amount of exposure. I could not help thinking some sort of bathing dress might be devised, for the personal exposure involved when the wooden cover is removed, as it was by the nurses and doctors who showed me the cases, would appear to be entirely unnecessary. I am bound, however, to say that in no case did the patients appear in the least to object to this uncovering. Bed-sores, spinal caries, septic wounds, and many similar cases are treated by the "perpetual bath." That is to say, the patient is kept in the bath until improvement shows—or the case proves hopeless.

The bedsore cases frequently remain in the bath for several weeks, and many of the doctors told me that in severe bedsore the water treatment is the only efficacious method of preventing pyæmia. I saw a case of myelitis and several other cases where the patient had remained in the bath for upwards of a year. The usual temperature of the water is 85 degrees to 92 degrees F., though this is modified to suit the condition, age, and temperament of the patient. Sometimes they are put at first in a bath of somewhat warmer temperature, and this is gradually cooled two or three degrees less, and the result carefully watched. For the first week the patients are made extremely unhappy by living constantly in water; indeed, I saw many women, new patients, weeping bitterly under the unwonted and unnatural custom of existence under water. But both doctors and nurses assured me that they soon become reconciled, and are quite happy after a time in their aquatic existence. Many of the patients looked extremely cheerful lying on their watery lounges eating their meals, writing letters, reading, or playing games of solitaire to solace them for the monotony of life in the restricted area of a bath-room.

I have seen the same treatment in Vienna, where the "perpetual bath" is much used in various skin diseases. In skin trouble treatment the patients are rarely kept in the water more than nine months, and all the bodily functions are performed in a perfectly normal way. Some doctors prefer the patient to be lifted up for the use of the bed-pan, while others allow all secretions to pass into the water. In such cases arrangement is made for the effectual drainage of the bath, and the used water is constantly flowing out and a fresh stream replacing it. In the Middle Ages in Germany baths were largely employed in the treatment of leprosy and other skin diseases. Since those days the treatment has undergone many modifications from the exaggerated German "water-cures," carried out by quack professors, and including the drinking of 10-15 glasses of cold water before breakfast and heroic open-air bathing at all seasons, down to the modified bath treatment adopted in modern German hospitals. Bath treatment should never be adopted save when prescribed by a medical man. And to carry it out effectually needs trained and skilled nursing supervision, for a patient undergoing the "perpetual treatment" needs careful watching, since the constant immersion in water has very differing effects on patients.

Among the many forms of water treatment I saw used in the German hospitals was the application of the chest pack, which differs somewhat from the English method. It is used very commonly in some varieties of asthma, empyema, chronic pleurisy, and other lung troubles. A piece of flannel about three yards long and three-quarters of a yard in width is folded lengthwise, done up in a roll, and soaked in hot, tepid, or cold water, according to instructions, and wrung out as dry as possible. The nurse, standing in front of her patient, applies the pack by placing the upper edge of one end close up under the left arm. Unrolling the flannel as she proceeds, she passes it across the front of the body and over the right shoulder, across the back and under the left arm, once more across the chest and under the right arm, round the back again and over the left shoulder. It is almost like a chest figure-of-eight. Fastening it with a safety-pin, she applies a dry flannel—slightly larger—in exactly the same way, and putting the patient between blankets, covers him up warmly and leaves him in the pack according to medical instructions. When the pack is taken off the skin surface should be bathed in tepid water, well dried, and the patient kept from taking a chill.

The wet girdle is another form of hydropathy which I noted is much used in the German treatment of uterine troubles, intestinal diseases, splenetic, dyspeptic, or liver troubles. In its application a piece of Turkish towelling rather over three yards in length is used. Half of the towel length is wet in cold or tepid water, according to prescription, and the wet end placed at one side; the towel is passed across the abdomen and under the back, and wound round till the towel length is used up, the dry half, of course, covering that which is wet and so protecting the bed. Care should be taken to have two folds of the wet part of the towel over the abdomen. Sometimes this is ordered to be worn through the night. In delicate and chilly patients it may be worn only for one or two hours out of the twenty-four; and, in patients with a feeble circulation, the wet part of the towel should be only on the abdomen, and not at the back. Applied to the back it sometimes produces faintness, and proves too stimulating to the spine. Space prevents me from describing the many and interesting varieties of the water-cure which I saw carried out in the German Hospital bath-houses, or from more than a mention of the peat and turf baths, where both these were made into poultices and smeared over the body, and a warm sponge bath given to remove the traces of such a novel form of bath.

The result of my bath-house tours proved not only extremely interesting from the point of view of the onlooker, but valuable from the professional standpoint; for in my tour of inspection I garnered up for future use many a useful wrinkle pertaining to baths and ablutions, the giving of which is a specially valuable art to the private nurse. I would strongly advise all peripatetic nurses whose wanderings and good fortune take them to Germany to take the opportunity thus afforded them of visiting the hospital bath-houses.

